# Application of the Workload Indicators of Staffing Need method to predict nursing human resources at a Family Health Service

**DOI:** 10.1590/1518-8345.1010.2683

**Published:** 2016-04-29

**Authors:** Daiana Bonfim, Ana Maria Laus, Ana Emilia Leal, Fernanda Maria Togeiro Fugulin, Raquel Rapone Gaidzinski

**Affiliations:** 1 PhD, Researcher, Escola de Enfermagem, Universidade de São Paulo, São Paulo, SP, Brazil; 2 PhD, Associate Professor, Escola de Enfermagem de Ribeirão Preto, Universidade de São Paulo, PAHO/WHO Collaborating Centre for Nursing Research Development, Ribeirão Preto, SP, Brazil; 3 MSc, RN, Unidade de Saúde Jardim Boa Vista, São Paulo, SP, Brazil; 4 PhD, Associate Professor, Escola de Enfermagem, Universidade de São Paulo, São Paulo, SP, Brazil; 5 PhD, Full Professor, Escola de Enfermagem, Universidade de São Paulo, São Paulo, SP, Brazil

**Keywords:** Nursing Staff, Primary Health Care, Workload

## Abstract

**Objective:**

verify the application of the Workload Indicators of Staffing Need method in the prediction of nursing human resources at a Family Health service.

**Method:**

descriptive and quantitative study, undertaken at a Family Health service in the city of São Paulo. The set of sequential operations recommended in the Workload Indicators of Staffing Need method was used: definition of the professional category, type of health service and calculation of Available Work Time; definition of workload components; identification of mean time for workload components; dimensioning of staff needs based on the method, application and interpretation of the data.

**Result:**

the workload proposed in the Workload Indicators of Staffing Need method to nursing technicians/auxiliary nurses was balanced with the number of professionals available at the Family Health service. The Workload Indicators of Staffing Need index amounted to 0.6 for nurses and 1.0 for nursing technicians/auxiliary nurses.

**Conclusion:**

the application of the Workload Indicators of Staffing Need method was relevant to identify the components of the nursing professionals' workload. Therefore, it is recommendable as a nursing staffing tool at Family Health services, contributing to the access and universal health coverage.

## Introduction

Human resources are one of the central pillars for access and universal health coverage, but represent a permanent challenge for many countries in Latin America, in view of disequilibria in their availability, composition, distribution and productivity, mainly in primary health care^(^ 1 ^)^.

In view of the essential role health professionals play in the protection, promotion and restoring of populations' health, it is fundamental for quantitative and qualitative planning and investment in the development of these professionals be done cautiously, so as to respond to the different and changeable health needs of the communities efficient and effectively^(^ 2 ^)^.

The planning process of health professionals looks for a balance between what is available in terms of market and what is necessary to guarantee attendance to the users' health demands^(^ 3 ^)^.

The dimensioning of nursing professionals, who representing the largest staff contingent in all health areas, has advanced in the discussion and enhancement of methods and parameters, mainly in hospital care. Nevertheless, in the Brazilian Primary Health Care context (PHC), specifically in the Family Health Strategy (FHS), few studies have addressed this theme.

A recent study presented data that provide a general view on the nursing interventions and activities at Family Health services (FHS) that have the potential to influence and improve the public policies regarding nursing staffing^(^ 4 ^)^.

This research was undertaken among 27,846 observations of the work by 34 baccalaureate nurses and 66 nursing technicians/auxiliary nurses, working at 27 FHS in the five geographic regions of Brazil, showing that the nurses and nursing technicians spend, on average, 70% of their work time of direct and indirect nursing care interventions^(^ 4 ^)^.

Although scientific evidence^(^ 5 ^)^ indicates a possible association between nursing staff density and maternal mortality, childhood mortality and immunization rates, predicting the number of professionals needed to attend to the users' needs at a FHS has not been easy.

The *Workload Indicators of Staffing Need* (WISN), a method the World Health Organization has proposed for staffing at a health institution^(^ 6 ^)^, signals great potential applicability at FHS and in a region's entire health service network.

The WISN departs from the workload, using activity (time) standards that are applicable to each workload component and to each professional's available time. This method provides results like the difference between the real and calculated number of nursing professionals, identifying the lack or surplus of a certain professional category^(^ 6 ^)^.

In view of the insufficient number of studies to support nursing staffing in PHC, the objective in this study is to verify the application of the WISN method in the prediction of nursing human resources at FHS.

## Method

In this descriptive study with a quantitative approach, a set of operations was used, recommended in the WISN, to calculate nursing professionals at a FHS in the city of São Paulo, Brazil, selected through a convenience sample, based on the criterion of being considered best primary health care practices.

This service was responsible for a territory of 5,639 families, equivalent to approximately 19,526 people, where care was offered from Mondays to Fridays (from 7 till 18 hours).

The work team consisted of six health teams, totaling six physicians, six nurses, 12 auxiliary nurses, 35 community health agents. Besides these professionals, there was a service manager, a nurse and a nursing technician for epidemiological surveillance and material sterilization, a physician for exclusive teaching and epidemiological surveillance activities, 10 administrative professionals, four dentists, one oral health aid, one oral health technician, one psychologist, one social worker, one occupational therapist, one pharmacist, three pharmaceutical technicians, three cleaning aids and one guard. The weekly workload was 40 hours.

A Social Health Organization (SO) managed the service through a comprehensive management contract based on the FHS. The main risks present in the coverage area were: mostly low-income living and work conditions, predominantly middle-class areas and two urbanized slums with illegal areas, drugs traffic, domestic violence, unemployment, risk of collapse and polluted creek. The most frequent health problems were arterial hypertension, diabetes mellitus and respiratory diseases. This service's status was due to the fact that one of its strengths was the union and participation of the attended population.

Following the steps described in the WISN method, the goal was to identify the core variables for nursing staffing.

## 1st Step: definition of professional category, type of health service and calculation of Available Working Time

The WISN method can be applied to all categories of health professionals and all types of services^(^ 6 ^)^. In this study, the nursing professionals from one FHS were analyzed.

The Available Working Time (AWT) refers to how long a health professional has available, in one year, to perform his job, discounting established (holidays and vacation) and unexpected (medical leave and training) days of absence. It can be expressed as days or hours per year^(^ 6 ^)^.

TTD = [A - (B + C + D + E ] x F

Where:

*AWT* = available work time per professional

*A* = number of possible workdays in one year (obtained by multiplying the number of weeks in one year (52) by the number of workdays in one week)

*B* = number of days of absence due to holidays in one year

*C* = number of days of absence due to vacation in one year 

*D* = number of days of absence due to medical leave in one year

*E* = number of days of absence due to other leaves, such as training, in one year

*F =* number of hours worked in one day.

## 2nd Step: definition of workload components

This step consists of defining the work interventions/activities that occupy most of the professionals' daily time. The most important interventions/activities on a health professional's daily agenda are considered as workload components, knowing that each component needs a specific amount of time^(^ 6 ^)^. The workload components corresponded to the interventions/activities the nursing professionals performed at the FHS, described in the data collection tool and classified according to the WISN method, as follows:

- Health service activities - developed by all members of a professional category, which identifies the particularity of the work and are generally registered;
- Support activities - complement the health activities, developed by all members of a professional category and generally are not registered;
- Additional activities - complement the health activities, developed by some members of a professional category and whose statistics are not registered regularly.

## 3rd Step: identification of mean time for workload components

Consists of the mean time needed for a trained, qualified and motivated professional to develop an intervention/activity with satisfactory competence/sill and attitude, according to the conditions and circumstance of each service^(^ 6 ^)^.

To identify the mean length of time the nursing staff spends to execute the interventions/activities that are the workload components, the work sampling technique was used, referring to the direct, structured, non-participatory observation of six nurses and 12 auxiliary nurses present at the service, during the eight-hour workday, every ten minutes, for five days (February 14^th^-18^th^ 2011).

The interventions/activities observed were registered in the data collection tool, consisting of nursing interventions/activities that were identified and validated for FHS^(^ 7 ^)^, work-related activities and personal activities, by two previously trained field observers, who accompanied an average nine professionals throughout the workday.

Intervention was considered as any treatment based on judgment and clinical knowledge, performed by a health professional to improve the results obtained by the users, family and community^(^ 8 ^)^; activity associated with activities of other professional categories, but which the health professional takes charge of and personal activity as the breaks needed in the workday to attend to the workers' physiological and personal communication needs.

The mean length of the interventions/activities was calculated per workload component. For the standard intervention/activity, that is, activities that are performed and registered routinely, the mean time was calculated based on the total time (in minutes) spent on each intervention/activity, divided by the number of users attended in the same period. In line with the WISN method, the survey of the number of users attended was based on available service statistics and reports. In this study, data for 2011 were used.

For the other two workload components, support interventions/activities and additional interventions/activities, whose statistics are not always available as they are not always registered, a mean length of time was calculated by adding up the frequencies (%) of the interventions observed plus the associated work activities, divided by the total number of observations in the period^(^ 6 ^)^, thus adding the adjustment factor the WISN method calls: Category Allowance Standard (CAS) and Individual Allowance Standard (IAS), numerically expressed as Category Allowance Factor (CAF) and Individual Allowance Factor (IAF), respectively.

To adapt to the proposed WISN terminology, the work-related activities and personal activities that were considered in the data collection tool were considered as support activities for the category and, as they represent a significant number of hours, they were allocated proportionately among the three workload components: standard, support and additional interventions/activities.

In this step, the lengths of time can be expressed as actual work time or as a percentage of the work time.

The percentage distributions and mean lengths of the interventions found in this study were used according to the professional category (nurse and nursing technician/auxiliary nurse) as nursing staffing parameters.

## 4th Step: staffing based on the method

For the purpose of staffing, the following procedure was adopted.

a) For the Health service activities : each workload component was divided by the AWT. This result showed the number of nursing staff needed per category to accomplish the workload component for the Health service activities at the FHS.
b) For the support interventions/activities, the result of item a) was multiplied by the category allowance factor. This procedure revealed the number of staff needed for all Health service activities and complementary interventions/activities for the category.
c) For the additional interventions/activities, the IAF was calculated and added up to the results of items a and b. Thus,

Staff need = Health service activities × CAF + IAF

## 5th Step: application and interpretation of the data in accordance with the WISN method

The difference between the number of staff available at the service and the staff needed was verified by analyzing the index between these two. When bordering on one (~1), the available staff is balanced with the staff demands for the workload at the service. An index superior to one (>1) evidences too much staff in relation to the workload and inferior to one (<1) that the current staff is insufficient to cope with the workload at the health service. Therefore, the lower the index, the greater the pressure at work^(^ 6 ^)^.

All participants were informed about the research objective, guaranteed anonymity, voluntary participation and signed the Informed Consent Form (ICF), with the approval of the Research Ethics Committee of the São Paulo Municipal Health Department, Process 249/09.

## Results

**Figure 1:**
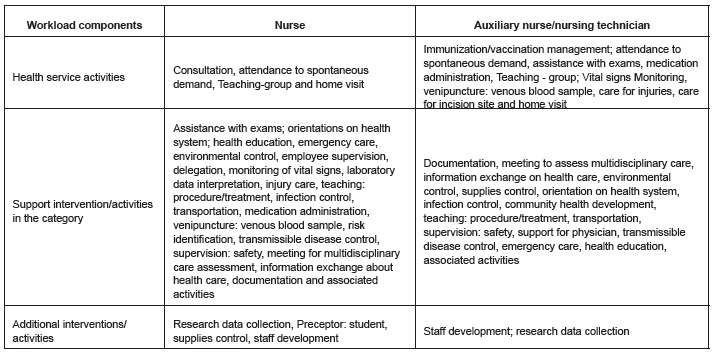
- Distribution of interventions/activities according to nursing workload components at FHS. São Paulo SP, Brazil, 2011

The nursing interventions/activities were classified according to the workload component, as demonstrated in Figure 1.

**Figure 2:**
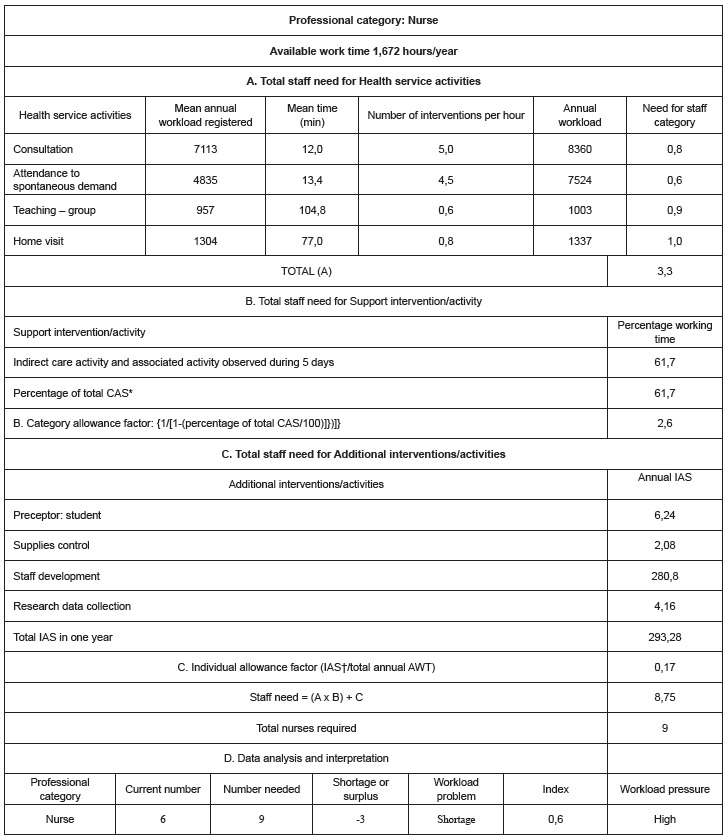
- Number of nurses required at a Family Health Service (FHS) according to WISN method. São Paulo, SP, Brazil, 2011

**Figure 3:**
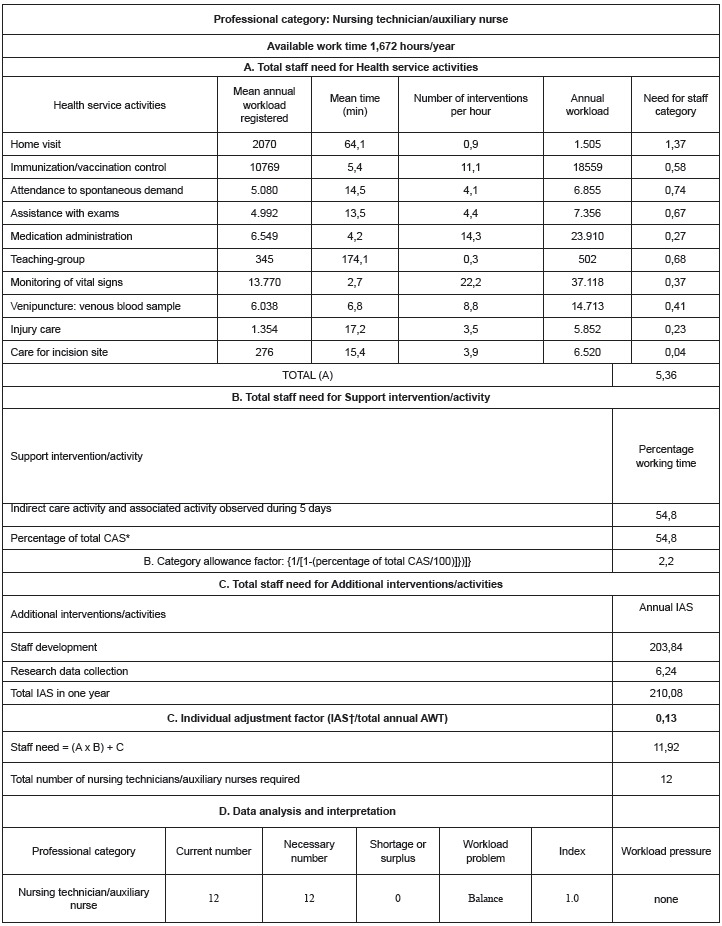
- Number of auxiliary nurses required at a Family Health Service according to WISN method. São Paulo, SP, Brazil, 2011

The number of nursing professionals required, according to the professional category, is demonstrated in Figures 2 and 3, which summarize the workload components, the steps proposed in the WISN method and the analysis and interpretation of the data.

*CAS: Category Adjustment Standard; †IAS: Individual Adjustment Standard

*CAS: Category Allowance Standard; †IAS: Individual Allowance Standard

## Discussion

This research identified the nursing staffing needs at a FHS in the city of São Paulo to attend to the care demands through the application of the WISN method.

The use of this method presupposes the availability of routinely stored data on the investigated professionals and services' workload. These statistics need to be updated, complete and consistent.

In that sense, a study appointed that the information system at the FHS contains insufficient spaces to report on the nursing team's work, showing the importance of qualifying the information systems developed to support the planning of nursing staffing needs^(^ 9 ^)^.

Therefore, the nursing care records provided to the users need to be systemized in reports or worksheets that permit monitoring the information for decision making, such as the service's annual production and data on professionals' expected and non-expected absences.

The AWT per professional category is calculated to find out how many work days or hours are available per year, representing more objective information on the reality of the service, which can facilitate negotiations with the health institutions' managers.

The results showed that there was disequilibrium between the nurse staffing needs identified through the WISN method and the existing nurse staff at the service analyzed. The length of the interventions/activities used in the calculations expressed the reality at the service. It is highlighted, however, that the mere use of the production data may not picture the needs of the population covered. Therefore, planning is needed to integrate the repressed demand. Therefore, the annual work load should be calculated based on this population's needs, considering the primary health care actions the Ministry of Health proposes^(^ 10 ^)^.

The direct observation and calculation of the mean lengths of the nursing professionals' interventions/activities performed were the differentials in this study, obtaining more precise time standards for the reality studied.

The introduction of the support interventions/activities, including both indirect care and work-related interventions, certainly constitutes a new aspect in staffing research, as it introduces activities the professionals generally refer to as time-consuming, but which can neither be demonstrated nor accounted for, as they lack formal registration, often making it difficult to measure the activity volume and their respective duration.

For the authors of the WISN method, adding workload components that are performed in a short period of time will make little difference for the final calculation of the number of professionals. Thus, this method highlights the importance of identifying the interventions/activities that truly affect the nursing professionals' workload in health care, with a view to elaborating planning that supports the capacity of the health system when attending to the population's needs.

In Brazil, the application of the WISN method at a FHS is a pioneering attempt to predict the number and quality of the nursing professionals.

Some experiences report on the successful application of the WISN method in different care realities.

In an Indonesian province, the midwives affirmed that the method was useful because it helped to focus their work time more clearly on key activities, besides permitting an analysis of their own work situation at the services^(11)^ . The WISN showed that the midwives were spending up to 50% of their time on activities not related to the midwife (elderly care, care for tuberculosis and malaria patients). Hence, the initial proposal that the number of midwives was insufficient for the category's specific workload, without the necessary clarification the WISN provides, could have resulted in an increased number of midwives instead of nurses^(^ 11 ^)^.

In provinces of Mozambique, the WISN was used to assessed its applicability, and thus expand the use of workload measures for the decision process. As a result, based on the staffing calculation, it was concluded that all health services had a lack of general clinicians, nurses and midwives. Therefore, the activities were performed within much less time than the minimum standard required, resulting in low quality. In addition, the distribution of nurses was unbalanced in the city of Nampula, with great disequilibrium between the hospital and the health services^(11)^ .

In a study developed in Namibia, the WISN results also appointed scarceness and inequality, showing that the nurses were distributed unequally among the different types of services and clearly deviated to the hospitals. Hence, the authors suggest that the health services use to WISN method to estimate the health professionals required for a range of needs and scenarios, including workers' adjustments in response to the implementation of new services, the decentralization or reconfiguration of primary care services^(^ 12 ^)^.

Evidences in the literature show that the use of a tool like WISN, when adapted to the local situation, improves the distribution of staff numbers among services, permits identifying the places where there is a lack of professionals and provides information support for planning, training and allocation at local, regional and national level^(^ 13 ^)^.

In terms of efficiency, the WISN can be considered a tool with potential to show ways to equate this distribution. Nevertheless, some limitations should be appointed in the WISN method, such as the precision determined by the exactness of the statistics. From that perspective, errors are almost always observed because of the insufficient registering of the workload, resulting in the underestimation of the staffing needs.

This study is limited by the fact that it was developed at a single FHS, making it impossible to generalize the obtained results, mainly related to the mean lengths of the nursing professionals' interventions/activities. Therefore, further research in different Brazilian realities will permit the identification of time parameters at the national and regional levels, making it possible to apply the WISN and assess the nursing professionals in the FHS in qualitative and quantitative terms.

## Conclusion

Different implementation contexts of primary health care in Brazil and the particularity of the FHS care model and the users' increasing demand make the effective planning of health professionals urgent.

The main contribution of this study, original in the Brazilian reality, rests in the application and assessment of the WISN method in the FHS, as an objective and systematic model for nursing staffing in PHC. Its application was relevant to identify the components of the nursing professionals' workload. Therefore, it is recommendable as a tool for the planning and qualitative and quantitative assessment of nursing professionals in FHS, so as to contribute to the access and universal coverage in health.
